# A double-blind randomized comparative clinical trial to evaluate the safety and efficacy of dendritic cell vaccine loaded with WT1 peptides (TLP0-001) in combination with S-1 in patients with advanced pancreatic cancer refractory to standard chemotherapy

**DOI:** 10.1186/s13063-019-3332-5

**Published:** 2019-04-27

**Authors:** Masahiro Katsuda, Motoki Miyazawa, Toshiyasu Ojima, Akio Katanuma, Kenichi Hakamada, Kentaro Sudo, Shingo Asahara, Itaru Endo, Makoto Ueno, Kazuo Hara, Suguru Yamada, Tsutomu Fujii, Sohei Satoi, Tatsuya Ioka, Masaichi Ohira, Takahiro Akahori, Masayuki Kitano, Hiroaki Nagano, Masayuki Furukawa, Tomohiko Adachi, Hiroki Yamaue

**Affiliations:** 1 Second Department of Surgery, Wakayama Medical University, School of Medicine, 811-1, Kimiidera, Wakayama, 641-8510 Japan; 2 Center for Gastroenterology, Teine-Keijinkai Hospital, Sapporo, Japan; 3 Department of Gastroenterological Surgery, Hirosaki University, Graduate School of Medicine, Aomori, Japan; 4 Division of Gastroenterology, Chiba Cancer Center, Chiba, Japan; 5 Department of Gastroenterology, Chiba Tokushukai Hospital, Chiba, Japan; 6 Department of Gastroenterological Surgery, Yokohama City University, Graduate School of Medicine, Yokohama, Japan; 7 Department of Gastroenterology, Hepatobiliary and Pancreatic Medical Oncology Division, Kanagawa Cancer Center, Yokohama, Japan; 8 Department of Gastroenterology, Aichi Cancer Center Hospital, Nagoya, Japan; 9 Department of Gastroenterological Surgery (Surgery II), Nagoya University, Graduate School of Medicine, Nagoya, Japan; 10 Department of Surgery and Science, Graduate School of Medicine and Pharmaceutical Sciences, University of Toyama, Toyama, Japan; 11 Department of Surgery, Kansai Medical University, Hirakata, Japan; 12 Department of Cancer Survey and Gastrointestinal Oncology, Osaka International Cancer Institute, Osaka, Japan; 13 Department of Surgical Oncology, Osaka City University Graduate School of Medicine, Osaka, Japan; 14 Department of Surgery, Nara Medical University, Nara, Japan; 15 Second Department of Internal Medicine, Wakayama Medical University, School of Medicine, Wakayama, Japan; 16 Department of Gastroenterological, Breast and Endocrine Surgery, Yamaguchi University, Graduate School of Medicine, Ube, Japan; 17 Department of Hepato-Biliary-Pancreatology, Kyushu Cancer Center, Fukuoka, Japan; 18 Department of Surgery, Nagasaki University Graduate School of Biomedical Sciences, Nagasaki, Japan

**Keywords:** Dendritic cells, Immunotherapy, Pancreatic cancer, S-1, Vaccine, Wilms’ tumor gene 1

## Abstract

**Background:**

Pancreatic cancer is a refractory malignancy, and the development of a new effective treatment strategy is needed. We generated a dendritic cell vaccine by culturing monocytes obtained by apheresis of blood from each patient, inducing their differentiation into dendritic cells, and pulsing with tumor antigen peptides. However, the clinical efficacy of the vaccine has not been established. We therefore decided to conduct an exploratory clinical trial of dendritic cell vaccine loaded with Wilms’ tumor gene 1 peptides (TLP0-001) as a potential new treatment for patients with advanced pancreatic cancer refractory to standard chemotherapy.

**Methods:**

This is an investigator-initiated, double-blind, comparative trial. The patients were allocated to two groups in a 1:1 ratio through a central registration by dynamic allocation. A total of 185 patients with inoperable or metastatic pancreatic cancer who were refractory or intolerant to standard primary chemotherapy with gemcitabine plus nab-paclitaxel will be allocated to secondary treatment either with placebo in combination with S-1 (the control group) or TLP0-001 in combination with S-1 (the investigational product group). The primary objective of this trial is to evaluate the safety and efficacy (as measured by overall survival) of the investigational product by comparing the two groups. This clinical trial will be performed in accordance with Japanese Good Clinical Practice guidelines.

**Discussion:**

Clinical trials of the standard regimen, including gemcitabine, for advanced pancreatic cancer are ongoing worldwide. However, a strategy for after the primary treatment has not been established. We therefore decided to conduct this study to evaluate the safety and efficacy of TLP0-001 as a secondary treatment for pancreatic cancer in anticipation of the approval of this new drug in Japan. This trial is conducted with full consideration of safety, as it is the first-in-human clinical trial of TLP0-001; thus, the trial will be conducted only at the Second Department of Surgery at Wakayama Medical University until the safety is confirmed by interim analysis. We plan to conduct a multicenter trial at 18 institutions in Japan after confirmation of the safety.

**Trial registration:**

University Hospital Medical Information Network Clinical Trials Registry, UMIN000027179. Registered on 9 April 2017.

**Electronic supplementary material:**

The online version of this article (10.1186/s13063-019-3332-5) contains supplementary material, which is available to authorized users.

## Background

Pancreatic cancer is a refractory malignancy with a poor prognosis. At the time of diagnosis, many patients have advanced disease and the tumor is often inoperable owing to its extension into adjacent large vessels or distant metastasis; in addition, recurrence is frequently observed in patients after radical surgery [1]. Therefore, the development of a new effective treatment strategy is needed.

In recent years, advances in molecular biology and immunology have resulted in the development of more successful immunotherapies for malignancies than was previously possible. Currently, immunotherapy using monoclonal antibodies directed against tumor antigens is a standard therapy for several types of cancer. Furthermore, immune checkpoint inhibitors have been established as a standard therapy for a variety of malignant tumors. In recent years, combination immunotherapy has also been developed. The effectiveness of sipuleucel-T (Provenge; Dendreon, Seal Beach, CA, USA) was demonstrated in asymptomatic or minimally symptomatic patients with prostate cancer resistant to hormone therapy [2], and it was approved as the world’s first dendritic cell vaccine therapy by the US Food and Drug Administration.

This trial focuses on a dendritic cell vaccine therapy in which tumor antigen-specific cytotoxic T lymphocytes (CTLs) are induced. We generated the dendritic cell vaccine by culturing a large number of monocytes obtained by apheresis of blood from each patient, inducing their differentiation into dendritic cells, and pulsing with tumor antigen peptides [3]. Dendritic cells are antigen-presenting cells and play the most important role in the induction of CTL. Clinical studies of dendritic cell vaccine therapies for various solid tumors and hematopoietic malignancies have been conducted in many medical institutions worldwide. However, their clinical effectiveness has not been established owing to the difficulty of reliable production of cells because of the complexity of the manufacturing method, difficulty in scale-up due to the autologous nature of the product, and difficulty in performing the appropriate randomized double-blind clinical trials.

This is an investigator-initiated, multicenter, randomized, double-blind, placebo-controlled, comparative trial of a dendritic cell vaccine loaded with Wilms’ tumor gene 1 (WT1) peptides (TLP0-001) as a potential vaccine therapy for patients with advanced pancreatic cancer refractory to standard chemotherapy. The WT1 gene is highly expressed specifically in pancreatic cancer [4]. Studies have reported that the WT1 protein, the gene product of WT1, is a promising target antigen for cancer vaccine therapy [4–15]. In addition, epitope peptides that are able to induce strong and specific CTLs against WT1 antigens have been identified, and a retrospective study of a dendritic cell vaccine loaded with WT1 peptides in combination with chemotherapy in patients with advanced pancreatic cancer suggested that survival was prolonged without serious adverse events [16]. Furthermore, other clinical studies of dendritic cell vaccine loaded with WT1 peptides in combination with chemotherapy in patients with advanced pancreatic cancer have confirmed the safety of administration of the vaccine and suggested that the antiproliferative effect on the tumor and the survival-promoting effect were greater in patients with a positive immune response [17, 18].

We are conducting this confirmatory clinical trial of a dendritic cell vaccine loaded with WT1 peptides (TLP0-001) as a novel treatment for inoperable or metastatic pancreatic cancer in anticipation of the approval of this new drug as a cellular and tissue-based product in Japan.

## Methods

### Aim

The primary objective of this clinical trial is to evaluate the safety and efficacy (as measured by overall survival [OS]) of TLP0–001 in patients with pancreatic cancer refractory or intolerant to standard therapy through a comparison of the control group (placebo in combination with S-1 [tegafur/gimeracil/oteracil]) and the investigational product group (TLP0-001 in combination with S-1).

### Study setting

This is an investigator-initiated, multicenter, randomized, double-blind, comparative trial. The patients are allocated to either the investigational product group or the control group in a 1:1 ratio through a central registration by dynamic allocation.

### Study design

**Fig. 1:**
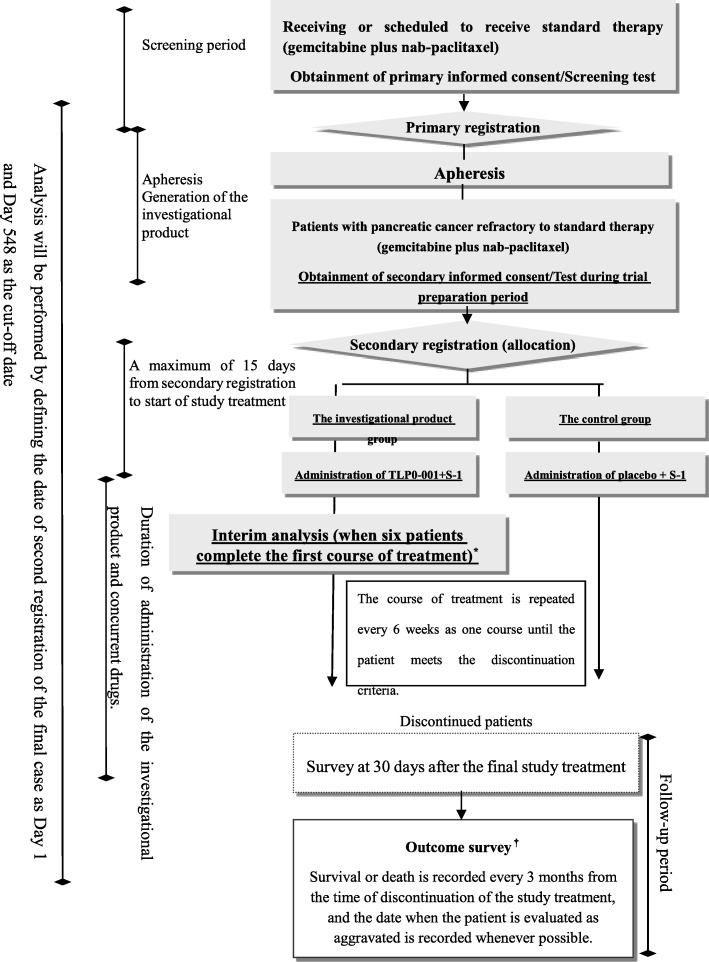
Schema of the trial. *An interim safety analysis for the period from the start of study treatment to the time when six patients complete the first course of treatment will be performed ^†^The outcome survey will be performed for the period from the date of secondary registration of the final case to Day 548

This clinical trial will be performed in accordance with the Japanese Good Clinical Practice guidelines. The schema of this trial is shown in Fig. 1. The Standard Protocol Items: Recommendations for Interventional Trials (SPIRIT) checklist is provided in Additional file 1. Primary informed consent will be obtained from patients with advanced or recurrent pancreatic cancer who are receiving or were scheduled to receive treatment including Gemzar (Eli Lilly and Company, Indianapolis, IN, USA) plus nab-paclitaxel and are willing to participate in this study. The patients who meet the eligibility criteria will be enrolled at primary registration. Subsequently, apheresis will be performed to collect cells for the production of the investigational product, and treatment with Gemzar plus nab-paclitaxel will be started or continued. When patients become refractory or intolerant to chemotherapy including Gemzar plus nab-paclitaxel, secondary informed consent for this study will be obtained. The patients who meet the eligibility criteria will be randomly allocated to either the investigational product group or the control group at secondary registration. Randomization will be performed using the Pocock-Simon minimization method, with allocation adjustment factors of institution and time of initial apheresis (before, during, or after primary treatment).

### Investigational and control products

#### Investigational product

For TLP0-001 injection the frozen product containing 1 × 10^7^ living dendritic cells per 1 mL solution will be thawed before use.

#### Control product

For placebo injection a frozen product which is indistinguishable from the investigational product but does not contain the active ingredient will be used. The frozen product will be thawed before use.

### Dosage and administration

The course of treatment with the investigational product in combination with S-1 will be repeated every 6 weeks as one course until the patient meets the discontinuation criteria.

For every course of treatment, S-1 will be administered for 4 weeks and then withdrawn for 2 weeks (i.e., administered every day from Day 1 to Day 28 and then withdrawn from Day 29 to Day 42). The investigational product will be administered every other week (three times, on Day 1, Day 15, and Day 29 of each course).

The investigational product will be manufactured by the organization supplying the investigational product (Tella Pharma Inc., Shinjuku, Tokyo) in accordance with Good Manufacturing Practice. The frozen investigational product will be thawed rapidly in a water bath for approximately 70 s at 37 °C at the time of administration. After the product is thawed, the contents will be directly aspirated with a syringe and administered within 1 h of thawing. The dose of the investigational product will be administered in a volume of 1000 μL. The product will be inoculated intradermally into the axilla and groin in fractional doses of 100 μL by using a syringe.

The initial dose of S-1 will be determined in accordance with the criteria presented in Table 1. S-1 will be administered in two equally divided doses (after breakfast and supper).

**Table 1:** Initial dose of S-1

Creatinine clearance^a^	Body surface area	Dose of S-1 (indicated as tegafur dose)
60 mL/min or more	Less than 1.25 m^2^	80 mg/day
	1.25 m^2^ or more, less than 1.5 m^2^	100 mg/day
	1.5 m^2^ or more	120 mg/day
Less than 60 mL/min	Less than 1.25 m^2^	50 mg/day
	1.25 m^2^ or more, less than 1.5 m^2^	80 mg/day
	1.5 m^2^ or more	100 mg/day

^a^Creatinine clearance will be estimated from the Cockcroft-Gault formula, although actual measurement values will be used if available

### Treatment discontinuation criteriad

The study treatment will be discontinued when a patient meets any of the following criteria during the period of administration of the investigational product:

1. A patient cannot start the first course of study treatment within 15 days after the secondary registration.
2. Aggravation of the primary disease (including clinical aggravation of the primary disease without evident tumor growth by imaging studies) is observed. However, study treatment may be continued if a principal investigator judges that study treatment would be beneficial even in patients judged as having progressive disease according to the Response Evaluation Criteria in Solid Tumors (RECIST) v1.1 Japanese version by the Japan Clinical Oncology Group (JCOG).
3. Diffuse alveolar damage pattern is seen on chest imaging. The presence of a diffuse alveolar damage pattern is confirmed through consultation with respiratory specialists or radiologists in the hospital or specialists in the assessment of pneumonitis.
4. A patient with a lung lesion has pleural effusion which a principal investigator (or co-investigator) judges as difficult to control.
5. When an increase in pleural effusion is seen in patients with carcinomatous pleurisy.
6. When an adverse event that requires discontinuation of the investigational product or S-1 occurs.
  - When a patient cannot start the next course of treatment within 14 days after the scheduled date in the second course or later phases (the scheduled date is 42 days after Day 1 of the previous course. Starting the course 2 weeks after the scheduled date on the same day of the week is allowed)
  - When an adverse event that meets the dose reduction criteria of S-1 occurs after the maximum reduction, and the attending doctor judges that discontinuation of the treatment is needed
  - When the attending doctor judges that discontinuation of the treatment is needed because of adverse events other than above
7. When administration of the investigational product is skipped twice in a row
8. When pregnancy is confirmed
9. When a patient requests to withdraw from the trial
10. When a patient withdraws his or her consent
11. When a patient has difficulty in continuing treatment because of moving, changing hospital, or otherwise being unable to attend
12. When a patient is found to be ineligible after registration

The study treatment will also be discontinued when a principal investigator judges that it is necessary for any other reason.

### Endpoints

#### Primary endpoint

The primary endpoint is OS.

#### Secondary endpoints

The secondary endpoints are:

- Progression-free survival (PFS) according to the RECIST v1.1 Japanese version by the JCOG (judgement by an investigator, central review)
- PFS according to the immune-related (ir) RECIST (central review)
- Cytoreductive effect according to the RECIST v1.1 Japanese version by the JCOG (judged by an investigator, central review)
- Cytoreductive effect according to the irRECIST (central review)

#### Endpoints regarding safety

Endpoints regarding safety are:

- Occurrence of adverse events
- Laboratory values, vital signs, 12-lead electrocardiogram, chest computed tomography (CT) examination

#### Endpoints at interim analysis

An interim analysis for safety will be performed when six patients in the investigational product group can be included in the analysis of endpoints after the start of study treatment, and the safety for the continuation of the trial will be evaluated by the Data and Safety Monitoring Committee. The trial will be stopped if dose-limiting toxicity (DLT), for which a causal relationship with the investigational product cannot be excluded, occurs in at least three of the six patients.

Patients subjected to the analysis of primary endpoints at interim analysis will be either of the following:

1. Patients who developed DLT, for which a causal relationship with the investigational product cannot be excluded, before the end of the first course of treatment
2. Patients who completed the first course of treatment with TLP0-001 in combination with S-1 without developing DLT.

#### Exploratory endpoints (arbitrary tests)

The following tests related to the exploratory endpoints are performed in specific trial sites where the tests are available. Informed consent is obtained from patients before performing any tests:

- WT1-specific CTL (enzyme-linked immuno spot, tetramer)
- Intestinal flora test (fecal examination)
- Quality of life survey (European Organization for Research and Treatment of Cancer QLQ-C30, EuroQol 5 dimension 5 level [EQ-5D-5 L])
- Investigation of tumor local environment
- Analysis of systemic milieu in study patients
- Analysis of the investigational product

### Eligibility criteria at second registration

#### Inclusion criteria

The inclusion criteria are as follows:

1. Having an invasive ductal pancreatic cancer (including locally advanced pancreatic cancer and recurrent pancreatic cancer) in which the diagnosis of adenocarcinoma or adenosquamous carcinoma was confirmed at the initial tissue diagnosis or cytological diagnosis, regardless of the presence or absence of measurable lesions
2. Patients assessed as refractory or intolerant after receiving therapy including gemcitabine plus nab-paclitaxel. Regardless of the implementation of other chemotherapy (except for fluoropyrimidine anticancer drugs) and radiation therapy
  - Refractory: Aggravation of the primary disease (including clinical aggravation of the primary disease in the absence of evident tumor growth by imaging studies) is observed after receiving therapy including gemcitabine plus nab-paclitaxel (including dose reduction and cessation of treatment)
  - Intolerant: A condition in which a patient cannot continue the treatment, for example, owing to drug adverse reactions
3. Must be a minimum of 20 years of age and a maximum of 79 years of age at the time of obtaining primary informed consent
4. Karnofsky Performance Status score of 80 or greater
5. Having the following human leucocyte antigen (HLA) allele types for both class I and class II:
  - Class I: HLA-A*24:02, HLA-A*02:01, or HLA-A*02:06
  - Class II: in HLA-DR, DRB1*04:05, DRB1*08:03, DRB1*15:01, or DRB1*15:02; in HLA-DP, DPB1*05:01 or DPB1*09:01
6. Life expectancy of greater than 3 months
7. Must meet all of the following criteria for test results during the trial preparation period; in addition, must not have received granulocyte colony-stimulating factor, erythropoietin product, blood product, or blood transfusion within 7 days before the test:
  - Leukocyte count between 3000/mm^3^ and 12,000/mm^3^
  - Neutrophil count of 1500/mm3 or greater
  - Hemoglobin level of 9.0 g/dL or greater
  - Platelet count of 100,000/mm3 or greater
  - Total bilirubin level of 2.0 mg/dL or lower (3.0 mg/dL or lower for patients undergoing treatment of obstructive jaundice)
  - Aspartate aminotransferase level of 150 IU/L or lower
  - Alanine aminotransferase level of 150 IU/L or lower
  - Serum creatinine level of 1.2 mg/dL or lower
  - Creatinine clearance is 50 mL/min or greater; creatinine clearance will be estimated by the Cockcroft-Gault formula, although actual measurement values will be used, if available
8. Able to take drugs orally
9. Must meet the following criteria during the period from the last day of the previous treatment to the start day of the trial, where the last day of previous treatment is defined as Day 1:
  - Anticancer drug: Day 15 or later
  - Anticancer drug unapproved in Japan: Day 29 or later
    In the case of antibody preparation, Day 57 or later
  - Radiation therapy: Day 29 or later
    Patients who received radiation to the administration sites (the entire axilla and groin) in this clinical trial are excluded
  - Laparotomy: Day 15 or later
  - Systemic administration of corticosteroids: Day 15 or later
10. Written informed consent is obtained from the patient.

#### Exclusion criteria

The exclusion criteria are as follows:

1. A history of treatment with fluoropyrimidine anticancer drugs. However, patients who underwent preoperative/postoperative adjuvant chemotherapy and have had no recurrence for at least half a year after the last day of administration of the drug can be registered
2. A history of receiving cancer immunotherapy including, but not limited to, activated lymphocyte therapy, dendritic cell therapy, cancer vaccine therapy, and immune checkpoint inhibitors
3. Having a double cancer (disease-free interval is 1 year or shorter, excluding the period of postoperative adjuvant chemotherapy). However, patients with intraepithelial cancer or intramucosal cancer lesions can be registered
4. A history or presence of interstitial pneumonia or pulmonary fibrosis, as confirmed by chest CT examination
5. A history of serious hypersensitivity to S-1 or drugs containing the ingredient
6. A history of hypersensitivity to OK-432, penicillin G, gentamicin, or streptomycin
7. A history of hypersensitivity to pig-derived ingredients or mouse-derived ingredients
8. A history of serious allergy including, but not limited to, severe asthma exacerbation and anaphylactic shock
9. Patients with watery diarrhea
10. Confirmed brain metastasis, or suspected from the clinical symptoms
11. Patients with pleural effusion, ascites, or cardiac effusion requiring puncture and drainage
12. Confirmed or suspected active infection
13. Hepatitis B surface antigen-positive, or hepatitis B virus DNA detected by real-time polymerase chain reaction
14. Positive test for hepatitis C virus antibody, human T cell leukemia virus type 1 antibody, human immunodeficiency virus antibody, syphilis spirochete, or parvovirus (where positivity for parvovirus is determined by a DNA test)
15. A severe mental disorder or neurological disorder
16. A poorly controlled (as indicated by Common Terminology Criteria for Adverse Events [CTCAE] Grade 3 or higher) heart disease, lung disease, renal disease, or liver disease
17. A CTCAE Grade 4 event (including laboratory abnormalities) or other poorly controlled (as indicated by CTCAE Grade 3 or higher) comorbidities
18. Continuation of flucytosine, phenytoin, or warfarin potassium required
19. Systemic administration of the following drugs required during the period of treatment with the investigational product:
  - Corticosteroids (continuous administration)
  - Immunosuppressants, immunostimulants
  - Erythropoietin products
20. An autoimmune disorder requiring treatment
21. Investigational products (at least five doses) cannot be generated using autologous blood from apheresis performed during the period from primary registration to obtaining secondary informed consent
22. Participation in other clinical trials or clinical studies (except for non-interventional trials)
23. Pregnancy or inability to discontinue breast feeding in the period starting from administration of the investigational product to 120 days after the final administration. The patient or his/her partner is unwilling to use the required methods of contraception (condom, pessary, intrauterine contraceptive device, embedded contraceptive device, spermicide, vasectomy, tubal ligation ) during the period starting from administration of the investigational product to 180 days after the final administration for men and to 120 days after the final administration for women
24. Others criteria judged as ineligible by a principal investigator.

### Sample size

A clinical study by Todaka et al. showed that median survival time of patients refractory to gemcitabine receiving S-1 monotherapy was 5.8 months [19]. Based on this result, the threshold median survival time was defined as 5.8 months. In contrast, a retrospective study by the organization supplying the investigational product (Tella Pharma Inc.) showed that the median survival time of 20 patients receiving dendritic cell vaccine in combination with S-1 as secondary treatment after receiving Gemzar or Gemzar plus S-1 was 8.1 months. However, in this clinical study, the day of starting dendritic cell vaccine therapy was defined as Day 1. Given that it takes approximately 4 weeks to generate dendritic cell vaccine after the collection of cells from patients, approximately 1 month had passed from the time when the patients became refractory to Gemzar or Gemzar plus S-1 to the time of starting the vaccine therapy. In this clinical study, the dendritic cell vaccine is generated during the primary treatment, and the dendritic cell vaccine therapy in combination with S-1 is started immediately after transition to the secondary treatment (this trial). We therefore believe that it is justified to add 1 month (the period required to generate the dendritic cell vaccine) to the median OS reported by Tella Pharma Inc. Accordingly, the expected median OS is set as 9.0 months. If the median OS is 5.8 months for the control group (placebo in combination with S-1) and 9.0 months for the investigational product group (TLP0-001 in combination with S-1), the expected hazard ratio (HR) for the investigational product group relative to the control group is calculated to be 0.644. A log-rank test is used to test the null hypothesis (the HR for the investigational product group relative to the control group is 1) against the one-sided alternative hypothesis (the HR is below 1). Given the assumption of a power of 80% or higher and a two-sided significance level of 0.05, the required minimum sample size was 87 individuals per group. Accordingly, the sample size was set as 90 individuals or more per group (i.e., a total of 185 individuals) with an assumption that a few patients would become ineligible for the trial.

### Statistical analysis

The primary population for efficacy analysis will be the full analysis set, defined as the patients who are administrated investigational product or placebo at least once. The primary endpoint is OS, defined as the time from date of secondary registration to the date of death from any cause. PFS is counted from the date of secondary registration to the date of death without progression, or of progression as confirmed by the Diagnostic Radiology Committee. OS and PFS will be compared using a stratified log-rank test with a two-sided alpha of 0.05 stratified by institution and time of initial apheresis (before, during, or after primary treatment). The HRs and 80% and 95% confidence intervals (CIs) will be estimated by the Cox proportional hazards model. Survival estimation will be also carried out using the Harrington-Fleming test, with the weight proportional to cumulative death probability. For the analysis of cytoreductive effect, adverse events, and side effects, categorical outcomes will be summarized using frequency and percentage for each arm and will be compared using Fisher’s exact method. The odds ratios and 95% CIs will also be estimated.

### Observation/test/survey schedule

Tests will be performed in accordance with the following schedule. The day on which study treatment is started is defined as Day 1.

#### Schedule of administration of the investigational product and combined drug/safety assessment

Tests are performed according to the schedule of administration of the investigational product and combined drug/safety assessment (Table 2).

**Table 2:**
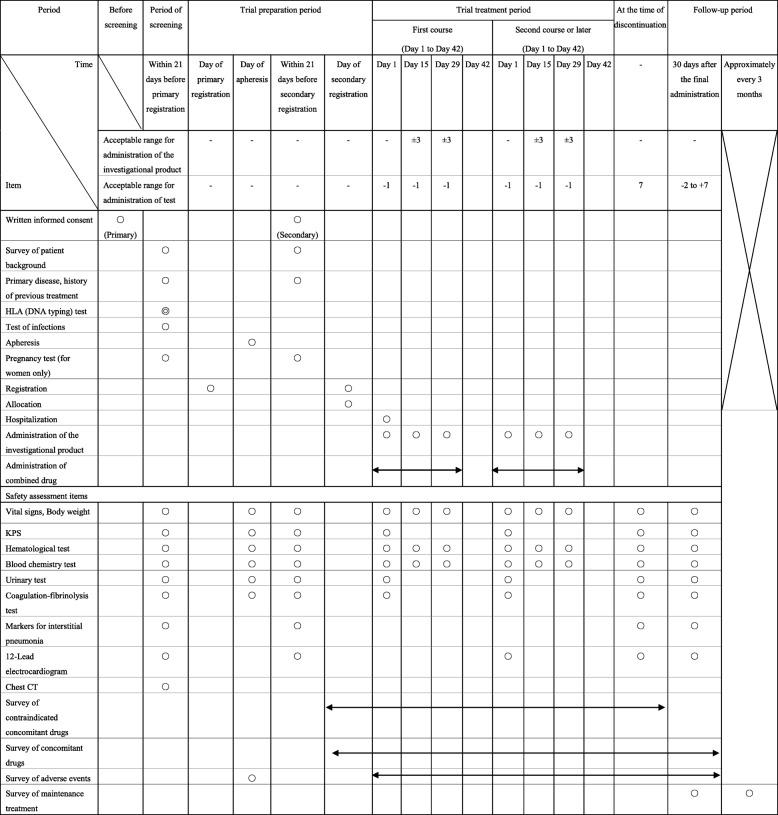
Schedule of administration of the investigational product and combined drug/safety assessment

*CT* computed tomography, *HLA* human leucocyte antigen, *KPS* Karnofsky Performance Status

○ Required

◎ The data obtained before informed consent was signed may be used, if available

#### Schedule of efficacy assessment/chest CT examination and exploratory assessments

Tests will be performed in accordance with the schedule of efficacy assessment/chest CT examination (Table 3) and exploratory assessments (Table 4).

**Table 3:**
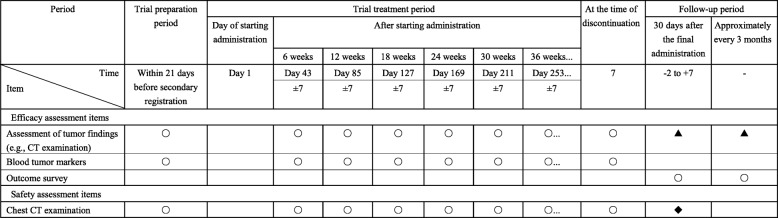
Schedule of efficacy assessment/chest CT examination

*CT* computed tomography

○ Required

▲ If a patient discontinues the study treatment for a reason other than aggravation of the primary disease, the date when the condition was aggravated is to be identified as much as possible (except for patients who started maintenance treatment). This is not required beyond 548 days after the secondary registration of the last case in this trial

◆ Performed if an adverse event for which a causal relationship with the investigational product cannot be excluded is observed

**Table 4:**
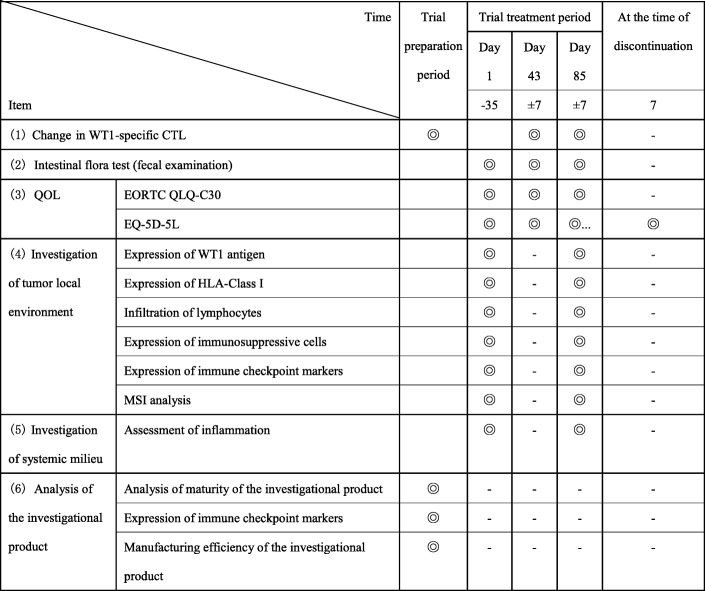
Schedule of exploratory assessments

*CTL* cytotoxic T lymphocyte, *EORTC QLQ-C30* European Organization for Research and Treatment of Cancer QLQ-C30, EQ-5D-5 L: EuroQol 5 dimensions 5 levels, *HLA* human leukocyte antigen, *MSI* microsatellite instability, *QOL* quality of life, *WT1* Wilms’ tumor gene 1

◎ Performed in specific trial sites where the tests are available. Informed consent is obtained from each patient before performing any tests

For these items, the day on which the first course of study treatment is started (Day 1) is used as the starting point.

## Discussion

Von Hoff et al. reported the results of the MPACT trial in initially treated patients with metastatic pancreatic cancer (861 cases), in which gemcitabine plus nab-paclitaxel therapy was significantly superior to gemcitabine monotherapy in terms of response rate (23% vs 7%, *p* < 0.001), PFS (5.5 months vs 3.7 months, *p* < 0.001), and OS as the primary endpoint (8.5 months vs 6.7 months, *p* < 0.001) [20]. Based on this and other evidence, gemcitabine plus nab-paclitaxel is currently the standard primary chemotherapy in Japan. Treatments including gemcitabine are usually selected for advanced pancreatic cancer worldwide; however, secondary or any subsequent treatments have not been established. The Clinical Practice Guidelines for Pancreatic Cancer (2016) state that “fluoropyrimidine-based chemotherapy is recommended as a secondary treatment for patients who received gemcitabine-based chemotherapy as a primary treatment, while gemcitabine-based chemotherapy is recommended as a secondary treatment for patients who received fluoropyrimidine-based chemotherapy as a primary treatment” [1]. In Japan, S-1 is frequently used as a secondary treatment. However, Nakai et al. studied the effectiveness of S-1 in patients who did not respond to gemcitabine and reported that the median survival time, including the period of treatment with gemcitabine, in patients who could receive S-1 as a second-line therapy was 11.3 months [21], which was not satisfactory. Todaka et al. also studied the effectiveness of S-1 in patients refractory to gemcitabine and reported that the median survival time was 5.8 months [19]. Therefore, the development of more effective treatment methods for patients with pancreatic cancer refractory to standard primary chemotherapy is needed. In this study, we developed a new regimen that combines TLP0-001 with S-1 for patients with advanced/recurrent pancreatic cancer refractory or intolerant to standard chemotherapy.

In a preliminary clinical trial conducted with administration of a dendritic cell vaccine loaded with WT1 peptides, 1 × 10^7^ living dendritic cells were administered intradermally every other week [17]. In this study, five of six patients (83.3%) confirmed the increased induction of WT1-specific CTLs after administration of dendritic cells. TLP0-001 is also considered to cause antitumor activity by inducing WT1-specific CTLs, and as a result of this preliminary clinical trial, the dosage and administration interval of TLP0-001 were determined. This is a placebo-controlled, double-blind, randomized trial to evaluate the superiority of the investigational product (TLP0-001 in combination with S-1) to the control product (placebo in combination with S-1) as the secondary treatment for advanced/recurrent pancreatic cancer, using OS as the endpoint. We are conducting this study in anticipation of the approval, including insurance coverage, of TLP0-001 as a secondary treatment for pancreatic cancer in Japan. This is the first-in-human clinical trial of TLP0-001. Therefore, the trial will be performed with full consideration of safety. To fully assure the safety of the subjects, an interim analysis will be performed when the first six patients of the investigational product group (TLP0-001 in combination with S-1) have completed the first course of treatment, and the safety for the continuation of the trial will be evaluated by the independent Data and Safety Monitoring Committee. The trial is only to be conducted at the Second Department of Surgery at Wakayama Medical University until the safety of the first course has been confirmed by the interim analysis. The procedure requires at least overnight hospitalization from Day 1 for observation.

We plan to conduct a multicenter trial at 18 institutions in Japan after the safety has been confirmed by interim analysis.

### Trial status

The Clinical Trial Notification was submitted to the Pharmaceuticals and Medical Devices Agency in January 2017, and recruitment began in March 14, 2017. The recruitment completion date is estimated to be March 2020. At the time of submission of this paper (December 2018), the protocol version is version 7.0 (November 21, 2018). This is a multicenter trial, for which the protocol will be approved by the Institutional Review Board of each trial site before the start of study treatment. Ethical approval has been confirmed from the Institutional Review Board at Wakayama Medical University (Protocol Identification Number: 1-28018A), and we will not begin recruiting at other centers in the trial until local ethical approval has been obtained.

## Additional file

Additional file 1: SPIRIT 2013 checklist: recommended items to address in a clinical trial protocol and related documents. (DOC 139 kb)

## Abbreviations

CT: Computed tomography
CTL: Cytotoxic T lymphocyte
DLT: Dose-limiting toxicity
JCOG: Japan Clinical Oncology Group
OS: Overall survival
PFS: Progression-free survival
RECIST: Response Evaluation Criteria in Solid Tumors
WT1: Wilms’ tumor gene 1
